# Editorial: Challenges, techniques and pitfalls in surgery: How far can we push the boundaries?

**DOI:** 10.3389/fonc.2022.1088759

**Published:** 2022-12-06

**Authors:** S. Cianci, F. Giovinazzo, G. Campagna, A. Ercoli

**Affiliations:** ^1^ Department of Human Pathology of Adult and Childhood “G. Barresi” Unit of Gynecology and Obstetrics, University of Messina, Messina, Italy; ^2^ General Surgery and Liver Transplantation Unit, Fondazione Policlinico Universitario Agostino Gemelli, Rome, Italy; ^3^ Department of Gynaecology and Obstetrics, Fondazione Policlinico Universitario “A. Gemelli”, Università Cattolica del Sacro Cuore, Roma, Italy

**Keywords:** surgery, endoscopy, gynecology, urology, surgical technologies

In the last decade, surgery to relevant was subjected to relevant improvement thanks technological innovations and consequent novel surgical techniques. The actual surgical panorama gained by the progress of minimally invasive surgery that has become the gold standard for several procedures historically performed with open access. This evolution involved different surgical fields, including general surgery, urology, gynaecology and many other specialities (1–3). Further evolution of minimally invasive surgery was represented by ultra-minimally invasive surgery, which is currently running through two different philosophies: the port size decreasing (3mm vs 5-10mm) (4) and the reduction of the incision numbers, represented by the single port approach (5). The latest innovations are represented by robotics. This technology allows for overcoming many limits of traditional endoscopy, even reducing the invasiveness.

Moreover, the different available platforms can actually allow for reduced costs (6). Furthermore, adjuvant therapies and new softwares can become fundamental in surgery. Therefore, these technologies could be useful even in different specialties as general surgery, surgical oncology, gynaecology, urology, etc. (7–12).

It was an very pleasure to serve as Guest Editors of the Research Topic of *Frontiers* entitled “*Challenges, Techniques and Pitfalls in Surgery: How Far Can We Push the Boundaries?*”. This Research Topic provides an overview of the last innovation in surgery and contributes to the field’s growth. All authors on the Research Topic contribute significantly to clinical and basic research advancements. Therefore, we present a collection of articles reported by authors from different specialities as general surgery to more specialistic surgical subspecialties. The article treated in this Research Topic could be useful for students, researchers and clinicians.

The Research Topic start with a case report by Wang et al. entitled “*Case Report: Gastric-Type Endocervical Adenocarcinoma Mimicking Submucosal Myoma Under Hysteroscopy*”, reporting a rare case of endocervical carcinoma. Concluding that GAS could be subject to misdiiagnosis.

The second article by Bao et al., entitled “Endoscopic Endonasal Supraoptic and Infraoptic Approaches for Complex “Parasuprasellar” Lesions: Surgical Anatomy, Technique Nuances, and Case Series” evaluates the use of the endoscopic technique for parasuprasellar lesions and reporting that these approaches could be effective in selected cases.

The third article by Wei et al., entitled “Clinical Application of Indocyanine Green Fluorescence Technology in Laparoscopic Radical Gastrectomy”, reports the outcomes of gastrectomy using new tracers reporting good outcomes in terms of operation time and intraoperative blood loss.

The fourth article by Xu et al., “Can a reresection be avoided after initial en bloc resection for high-risk non-muscle invasive bladder cancer? A systematic review and meta-analysis”, was a meta-analysis related to the surgery options for bladder cancer that in coclusion reported good oncologic and post-operative outcomes. The fifth article by Campagna et al., entitled “Laparoscopic High Uterosacral Ligament Suspension vs Laparoscopic Sacral Colpopexy for Pelvic Organ Prolapse: A Case-Control Study” was focused on different surgical approaches for pelvic organ prolapse concluding that both techniques are safe, feasible, and effective. The sixth article by Peng et al., entitled “The Transumbilical Laparoendoscopic Single-Site Extraperitoneal Approach for Pelvic and Para-Aortic Lymphadenectomy: A Technique Note and Feasibility Study” investigated the use of single incision surgery for lymphadenectomy reporting the feasibility of the technique. The seventh article by Santullo et al. entitled “The Road to Technical Proficiency in Cytoreductive Surgery for Peritoneal Carcinomatosis: Risk-Adjusted Cumulative Summation Analysis” reported a model aimed to improve the surgical outcomes for cytoreductive surgery. The eighth article by Cianci et al., entitled “Different Surgical Approaches for Early-Stage Ovarian Cancer Staging. A Large Monocentric Experience”, investigated advantages ans disadvantages of different surgical approaches for ovarian cancer treatment even from an oncological point of view. The ninth article by Li et al., “Toward Exempting from Sentinel Lymph Node Biopsy in T1 Breast Cancer Patients: A Retrospective Study”, reported exciting data on the sentinel lymph node for breast cancer. The tenth article by Zhang et al., “A Scientometric Analysis and Visualization Discovery of Enhanced Recovery After Surgery”, which applied the analysis to ERAS guidelines. The eleventh article by Zhao et al., entitled “Application Status and Prospects of Artificial Intelligence in Peptic Ulcers”, reported an article related to the clinical use of artificial intelligence. The twelfth article by Spalthoff et al., “Time is crucial in malignant tumour cases: Speeding up the process of patient-specific implant creation, ” focused on the time importance for patients implant creation affirming the importance of procedural standardization.

The thirteenth article by Zhu et al. entitled “Laparoscopic Subtotal Gastrectomy and Sigmoidectomy Combined With Natural Orifice Specimen Extraction Surgery (NOSES) for Synchronous Gastric Cancer and Sigmoid Colon Cancer: A Case Report” was a case report focused on the use of natural orifice used for specimen extraction reporting good outcomes.

The fourteenth article by Xiong et al., entitled “Partial Nephrectomy Versus Radical Nephrectomy for Endophytic Renal Tumors: Comparison of Operative, Functional, and Oncological Outcomes by Propensity Score Matching Analysis”, studied the outcomes of different procedures for renal tumours surgery. The fifteenth article by Tang et al., entitled “Preliminary Analysis of Safety and Feasibility of a Single-Hole Laparoscopic Myomectomy via an Abdominal Scar Approach, “ focused on a single port approach for gynecologic surgery demonstrating the feasibility.

The sixteenth article by Zuo et al., entitled “O-arm-guided percutaneous microwave ablation and cementoplasty for the treatment of pelvic acetabulum bone metastasis”, reported exciting data on a new surgical strategy for bone surgery based on microwave instrument.

The seventeenth article by Abdullah et al., entitled “Laparoscopic retroperitoneal resection of the duodenal gastrointestinal stromal tumours in neurofibromatosis type 1; Case Report and literature review” reported an article focused on endoscopic treatment of gastrointestinal tumours reporting good surgical outcomes.

The eighteenth, by Wang et al., entitled “Effect of fetoscopic laser surgery on the placental characteristics and birth-weight discordance of twins with twin-to-twin transfusion syndrome”, investigated the use of laser technology for fetal surgery.

The nineteenth article by Ishikawa and Shozu, entitled “Modified Leak-Proof Puncture Technique for the Aspiration of Giant Ovarian Cysts by Instantly Mounting a Plastic Wrap and Gauze with Cyanoacrylates: A Retrospective Observational Study”, reported a technique for aspiration of giant cysts concluding the feasibility of the technique in selected cases.

The last article by Catena et al., entitled “Fertility-sparing treatment for endometrial cancer and atypical endometrial hyperplasia in patients with Lynch Syndrome: Molecular diagnosis after immunohistochemistry of MMR proteins” was focused on fertility sparring treatment for syndromic patients giving relevant indications for these cases.

We appreciated the effort of all authors put in their articles, which significantly contribute to the scientific panorama.

## Author contributions

CS, GF, CG, EA contributed to conception and design of the study equally. CS wrote the first draft of the manuscript. GF, CG, EA contribute equally at the final version of the manuscript. All authors contributed to manuscript revision, read, and approved the submitted version.

## References

[1] Borzellino G Ruzzenente A Minicozzi AM Giovinazzo F Pedrazzani C Guglielmi A . Laparoscopic hepatic resection. Surg Endosc (2006) 20(5):787–90. doi: 10.1007/s00464-004-2186-3 16544083

[2] McNeill SA Tolley DA . Laparoscopy in urology: indications and training. BJU Int (2002) 89(3):169–73. doi: 10.1046/j.1464-4096.2001.01891.x 11856092

[3] Cianci S Capozzi VA Rosati A Rumolo V Corrado G Uccella S . Different surgical approaches for early-stage ovarian cancer staging. a Large monocentric experience. Front Med (Lausanne) (2022) 9:880681. doi: 10.3389/fmed.2022.880681 35547212 PMC9081786

[4] Rossitto C Cianci S Gueli Alletti S Perrone E Pizzacalla S . Scambia G laparoscopic, minilaparoscopic, single-port and percutaneous hysterectomy: Comparison of perioperative outcomes of minimally invasive approaches in gynecologic surgery. Eur J Obstet Gynecol Reprod Biol (2017), 216:125–129. doi: 10.1016/j.ejogrb.2017.07.026 28753500

[5] Zeinab MA Beksac AT Ferguson E Kaviani A Moschovas MC Joseph J . Single-port extraperitoneal and transperitoneal radical prostatectomy: A multi-institutional propensity-score matched study. Urology (2022), S0090–4295(22)00874-3. doi: 10.1016/j.urology.2022.10.001 36244472

[6] Cianci S Rosati A Rumolo V Gueli Alletti S Gallotta V Turco LC . Robotic single-port platform in general, urologic, and gynecologic surgeries: A systematic review of the literature and meta-analysis. World J Surg (2019) 43(10):2401–19. doi: 10.1007/s00268-019-05049-0 31187247

[7] Shetty B Fernandes R Rodrigues AP Chengoden R Bhattacharya S Lakshmanna K . Skin lesion classification of dermoscopic images using machine learning and convolutional neural network. Sci Rep (2022) 12(1):18134. doi: 10.1038/s41598-022-22644-9 36307467 PMC9616944

[8] Ilyas M . Artificial intelligence in cancer pathology-hope or hype? Lancet Digit Health (2022) 4(11):e766–7. doi: 10.1016/S2589-7500(22)00193-5 36307189

[9] Campagna G Panico G Caramazza D Anchora LP Parello A Gallucci V . Laparoscopic sacrocolpopexy plus ventral rectopexy as combined treatment for multicompartment pelvic organ prolapse. Tech Coloproctol (2020) 24(6):573–84. doi: 10.1007/s10151-020-02199-5 32285229

[10] Panico G Campagna G Caramazza D Amato N Ercoli A Scambia G . Laparoscopic high uterosacral ligament suspension: an alternative route for a traditional technique. Int Urogynecol J (2018) 29(8):1227–9. doi: 10.1007/s00192-018-3588-4 29500517

[11] Campagna G Panico G Morciano A Vacca L Anchora LP Gallucci V . Laparoscopic supracervical hysterectomy and sacral colpopexy for pelvic organ prolapse with percutaneous surgical system: Results from a pilot study. eur J obstet gynecol reprod biol. European Journal of Obstetrics and Gynecology and Reproductive Biology (2018) 221:160–5. doi: 10.1016/j.ejogrb.2017.12.043 29310041

[12] Peters BS Armijo PR Krause C Choudhury SA Oleynikov D . Review of emerging surgical robotic technology. Surg Endosc (2018) 32(4):1636–55. doi: 10.1007/s00464-018-6079-2 29442240

